# Risk of Recall Among Medical Devices Undergoing US Food and Drug Administration 510(k) Clearance and Premarket Approval, 2008-2017

**DOI:** 10.1001/jamanetworkopen.2021.7274

**Published:** 2021-05-06

**Authors:** Jonathan R. Dubin, Stephen D. Simon, Kirsten Norrell, Jacob Perera, Jacob Gowen, Akin Cil

**Affiliations:** 1 Department of Orthopedic Surgery, University of Missouri–Kansas City, Kansas City; 2 Department of Orthopedic Surgery, Truman Medical Center, Kansas City, Missouri; 3 Department of Medical and Health Informatics, University of Missouri–Kansas City, Kansas City

## Abstract

**Importance:**

The US Food and Drug Administration (FDA) uses 510(k) clearance and premarket approval (PMA) pathways to ensure device safety before marketing. Premarket approval evaluates high-risk medical devices and requires clinical trials, whereas 510(k) clearance evaluates moderate-risk devices and relies on benchtop (nonclinical and biomechanical) and descriptive data. Existing literature suggests that the clinical trials required by PMA are associated with reduced risk of recall compared with devices granted 510(k) clearance. Several investigators have found weaknesses in pivotal PMA trials, raising safety concerns. Furthermore, methodological factors may have led to a previous underestimation of recall risk for devices with PMA.

**Objectives:**

To compare risk of recall and high-risk recall between devices that received 510(k) clearance and those that received PMA and to compare the risk of recall between devices for medical specialties.

**Design, Setting, and Participants:**

This cohort study compared devices with 510(k) clearance vs those with PMA that reached the market between January 1, 2008, and December 31, 2017. Two- to 12-year follow-up was obtained from the FDA’s 510(k) and PMA medical device database. Orthopedic surgery was chosen arbitrarily as the reference category for analysis between specialties because no baseline exists. Statistical analysis was performed from February 1 to November 1, 2020.

**Main Outcomes and Measures:**

The FDA issues recalls for safety concerns. These recalls are stratified into class I, II, and III, with class I representing high-risk issues for serious harm or death. The main outcome was the hazard ratio of any recall and class I recall between devices with PMA and those with 510(k) clearance. The secondary outcome was the recall hazard ratio between specialties with respect to the reference category. A single Cox proportional hazards regression model evaluating the association of medical specialty and FDA approval pathway with the risk of recall was performed.

**Results:**

During the study period, 28 246 devices received 510(k) clearance and 310 devices (10.7%) received PMA; 3012 devices (10.7%) with 510(k) clearance and 84 devices (27.1%) with PMA were recalled. A total of 216 devices (0.8%) with 510(k) clearance and 16 devices (5.2%) with PMA had class I recalls. Devices with PMA compared with those with 510(k) clearance had a hazard ratio for recall of 2.74 (95% CI, 2.19-3.44; *P* < .001) and a hazard ratio for high-risk recall of 7.30 (95% CI, 4.39-12.13; *P* < .001). Only radiologic devices were associated with an increased risk of recall (hazard ratio, 1.57; 95% CI, 1.32-1.87; *P* < .001), whereas 6 specialties were assocated with a decreased risk compared with the orthopedic reference category: general and plastic surgery, otolaryngology, obstetrics and gynecology, physical medicine, hematology, and general hospital.

**Conclusions and Relevance:**

This study suggests that high-risk medical devices approved via PMA are associated with a greater risk of recall than previously reported. Most recalls are for devices with 510(k) clearance, also raising safety concerns. Strengthening postmarketing surveillance strategies and pivotal trials may improve device safety.

## Key Points

**Question:**

What is the risk of recall and high-risk recall for devices undergoing US Food and Drug Administration (FDA) 510(k) clearance compared with premarket approval (PMA)?

**Findings:**

In this cohort study using the FDA’s 510(k) and PMA medical device database, 28 556 devices were reviewed. Although 97% of recalled devices had received 510(k) clearance, devices with PMA had 2.7 times the hazard of recall and 7.3 times the hazard of high-risk recall compared with devices with 510(k) clearance.

**Meaning:**

This study suggests that, despite the requirement of clinical trials, high-risk devices approved via PMA were associated with greater safety concerns than previously reported; in addition, most recalls are for 510(k) devices, raising safety issues.

## Teaser

This cohort study compares risk of recall and high-risk recall between medical devices that received US Food and Drug Administration 510(k) clearance and those that received premarket approval.

## Introduction

Understanding the regulatory process used by the US Food and Drug Administration (FDA) to ensure the safety and effectiveness of medical devices is essential, from the policy level down to an individual surgeon contemplating the choice of an implant for a patient. Several highly publicized device failures have renewed calls for regulatory change.^1,2,3,4,5^ In an attempt to objectively assess device safety, multiple authors have investigated the risk of recall for devices undergoing 510(k) clearance compared with the more rigorous premarket approval (PMA).^6,7,8,9,10,11,12^

Medical device regulation has been described previously.^13,14^ In brief, the FDA stratifies devices according to risk: class I is minimal (eg, tongue depressors), class II is moderate (eg, tibia nails and powered wheelchairs), and class III is high (eg, implantable pacemakers). The pathway that a novel device takes through the FDA to reach the market depends largely on this classification. Class III devices require a PMA, whereas class II devices typically undergo 510(k) clearance. Class I products are usually exempt from formal testing.^13,14,15^

A fundamental difference between PMA and 510(k) clearance is in the burden of proof required. A successful PMA application must prove a reasonable assurance of safety and effectiveness, whereas the 510(k) application must show only substantial equivalence to another device already on the market. To achieve the former requires a clinical trial, whereas the latter frequently relies on benchtop (nonclinical and biomechanical) tests and descriptive analysis.^16,17,18^

Investigators have found reason for concern in both approval pathways. Dhruva et al^19^ found that only 27% of the studies used to support cardiovascular devices with PMA were randomized, and just 14% were blinded in any way. Barker et al^20^ reported similar concerns regarding study strength in pivotal trials for orthopedic devices. Despite these findings, most authors reporting on device safety have expressed greater concerns regarding 510(k) clearance because of the frequent lack of clinical evidence.^6,7,8,9,10,11,12,20,21,22,23^ In a study by Zuckerman et al,^7^ 71% of high-risk recalls were for devices with 510(k) clearance. Day et al^6^ found that devices with 510(k) clearance were 11.5 times more likely to be recalled than those with PMA, whereas Somberg et al^11^ reported that devices with 510(k) clearance were twice as likely as those with PMA to have a high-risk recall. This trend has been demonstrated in the obstetric, otolaryngologic, and radiologic literature.^8,9,10,12^ In 2011, the Institute of Medicine completed an evaluation of the 510(k) clearance process over its 35 years and recommended it be phased out.^22^

To our knowledge, no peer-reviewed literature exists that accurately establishes the risk of recall for a device approved by 510(k) clearance or PMA. Nearly all of the existing literature retrospectively analyzes only the subset of recalled devices. This approach is problematic for multiple reasons: (1) the volume of devices with 510(k) clearance on the market is vastly greater than that of devices with PMA, so one would expect proportionately more recalls of devices with 510(k) clearance; (2) previous studies have not accounted for the duration of time the device is on the market and exposed to the risk of recall; and (3) devices can be recalled multiple times, so a single problematic device can disproportionately weigh on the results. Two authors^6,11^ have attempted to account for some of these issues; however, methodological concerns still hamper the applicability of their results. Day et al^6^ looked only at recalls within the top 20 companies, which they divided into various medical specialties based on the supposed field the company most commonly serves. For instance, in their analysis, all Depuy and Medtronic devices were considered “orthopedics,” demonstrating the high level of subjectivity in this approach. Somberg et al^11^ considered only high-risk recalls and did not account for the time each device was on the market.

The present study represents the largest to date, to our knowledge, that analyzes both all recalls and high-risk recalls. The goal is to establish an accurate assessment of the risk of recall and high-risk recall for devices with 510(k) clearance and those with PMA by performing a time-to-event analysis and to subsequently test the hypothesis that devices with 510(k) clearance pose a greater risk of recall than those with PMA. Secondary aims included comparing the risk of recall between medical specialties and evaluating the total number of recall events to address the impact of devices with multiple recalls.

## Methods

### Device Inclusion

From the FDA’s 510(k) database, all devices that received clearance between January 1, 2008, and December 31, 2017, were downloaded to a Microsoft Excel, version 16.0 (Microsoft Corp) spreadsheet.^24^ The filter option “Panel” was set to a specific FDA medical specialty (eg, cardiovascular or orthopedics) to classify each device into 1 of 19 specialties. This classification yielded 29 898 devices; however, a duplicate check in Excel found 1652 devices listed under 2 separate specialty panels. These devices were individually searched on the database using their FDA-given 510(k) number and then assigned to the specialty designated by the FDA as “510(k) Review Panel,” leaving a total of 28 246 unique devices with 510(k) clearance. This cohort study followed the Strengthening the Reporting of Observational Studies in Epidemiology (STROBE) reporting guideline. The University of Missouri–Kansas City institutional review board deemed this study exempt from institutional review board approval because it does not involve human participants.

The PMA database was similarly queried, but with adding the filter “Supplement Type” set to “Originals Only.”^25^ Manufacturers can submit PMA supplements for devices with existing PMA for several reasons, including labeling changes, device modifications, and expanding indications. Supplements do not exist for devices with 510(k) clearance, and significant changes require a new 510(k) application.^26^ Although valid criticisms have been raised about the safety of PMA supplements and how their accumulation over time can substantially alter the original device, the FDA does not treat supplements as unique devices, considering them only modifications to the original device.^26,27,28^ As such, in the database, a recall is listed only under the original device’s PMA number, even if the cause was from a change introduced by a supplement, as was the case with the Medtronic Sprint Fidelis Lead.^29,30^ To stay consistent with FDA methods, we included only the original PMA in the accounting of total devices with PMA.

### Data Collection

The end point of the study was December 31, 2019, providing 2 to 12 years of follow-up for the devices on the market. Owing to the size of the data set, data were abstracted repeatedly throughout January 2020 by clicking on the link for each device in the results from the searches described. Further relevant information for recalled devices, including recall dates and type, was then collected from the link for each individual device. The FDA codifies recalls according to the severity of potential harm, with class I being the highest, class II being moderate, and class III being the lowest.^31^ Recalls can be issued for multiple parts of the same device; to prevent overcounting, recalls on the same date were counted as 1 recall.

### Statistical Analysis

Statistical analysis was performed from February 1 to November 1, 2020. Microsoft Excel, version 16.0 was used for descriptive analysis, and R, version 4.0.0 (R Group for Statistical Consulting) was used for statistical analysis. A single Cox proportional hazards regression model that evaluated the association of medical specialty and FDA approval pathway with the risk of recall was performed. Although selection of a reference category for the analysis between specialties is arbitrary because no true baseline exists, orthopedic surgery was chosen because the category contained the largest number of devices, and the overall percentage of recalled devices appeared similar to the group of all devices as a whole. The *P* values and 95% CIs for comparing each device specialty with orthopedics have been adjusted for multiple comparisons using the Bonferroni correction. All tests were 2-sided using an α of .05. A second Cox proportional hazards regression model was created to compare high-risk recalls (class I) between devices with PMA and devices with 510(k) clearance.

The Cox proportional hazards regression assumption was examined using Schoenfeld residuals and the complementary log-log plot. Several very minor departures from proportional hazards were seen, but these departures were limited to very early or very late time ranges.

## Results

### Overview by Devices

**Figure 1.**
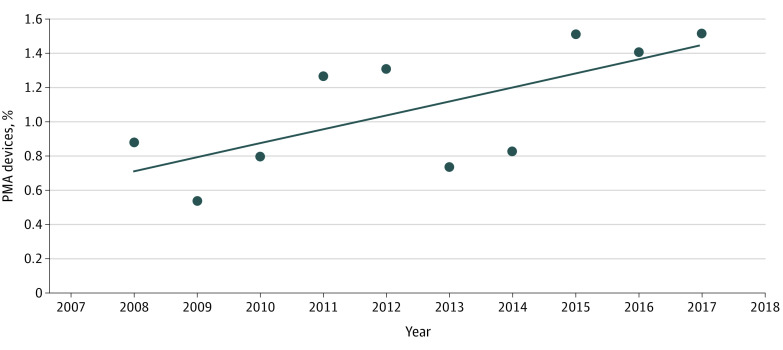
Devices That Received Premarket Approval (PMA) per Year as Percentage of All Devices

The FDA cleared a mean of 2825 devices (95% CI, 2733-2917 devices) per year by 510(k) compared with a mean of 31 devices (95% CI, 24-38 devices) approved per year under PMA. A total of 28 556 devices came to market during the 10-year study period; while the proportion of devices with PMA did increase from 0.7% to 1.5%, overall, only 310 devices (1.1%) were approved through the PMA process, and 28 246 devices received 510(k) clearance (Figure 1). There were 84 devices with PMA (27.1%) recalled vs 3012 devices with 510(k) clearance (10.7%). A total of 216 devices with 510(k) clearance (0.8%) underwent a class I recall compared with 16 devices with PMA (5.2%) (Table 1).

**Table 1.** Overview of Device Recalls and Total Recall Events^a^

Approval pathway	Total No. of devices	Total No. (%)		Total No. of recalls	No. (%) of devices with multiple recalls	Total No. of class I recall events (% of total recalls)
		Recalled devices	Devices with class I recall			
510(k)	28 246	3012 (10.7)	216 (0.8)	5218	960 (3.4)	269 (5.2)
PMA	310	84 (27.1)	16 (5.2)	144	26 (8.4)	28 (19.4)

Abbreviation: PMA, premarket approval.

^a^ Total number of devices cleared or approved for each pathway from January 1, 2008, to December 31, 2017. The recall analysis was carried out to December 31, 2019, to provide a follow-up of 2 to 12 years.

### Overview by Recalls

There were 5362 recall events among all devices included in the study. Of these, 97.3% (5218) were for devices with 510(k) approval, and 2.7% (144) were for PMA devices. The greater number of total recalls compared with devices recalled is secondary to the frequency of devices undergoing multiple recalls. Of the 84 devices with PMA that were recalled, 31.0% (26) had multiple recall events. Similarly, of the 3012 devices with 510(k) clearance that were recalled, 31.9% (960) experienced multiple recalls.

### Risk of Recall: Devices With 510(k) Clearance vs PMA

**Figure 2.**
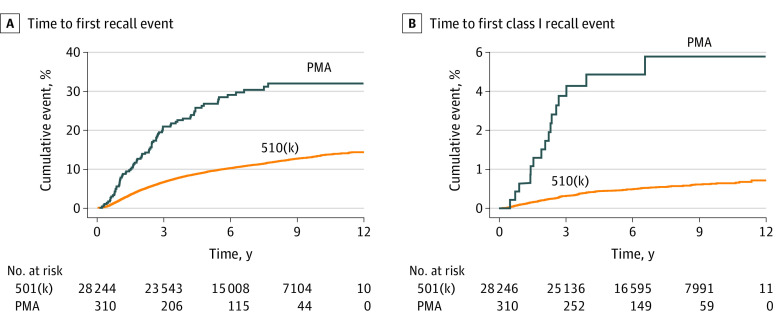
Time to Recall Events A, Time to first recall event, devices with premarket approval (PMA) vs 510(k) clearance. B, Time to first class I recall event, devices with PMA vs 510(k) clearance.

Compared with devices with 510(k) clearance, the hazard ratio for recall of devices with PMA was 2.74 (95% CI, 2.19-3.44; *P* < .001). For only class I recalls, the hazard ratio for devices with PMA increased to 7.30 (95% CI, 4.39-12.13; *P* < .001) (Figure 2A and B). At 9 years, the Kaplan-Meier curves show that the probability of recall for devices with 510(k) clearance is 12.6% (95% CI, 12.1%-13.2%) compared with 32.0% (95% CI, 23.1-40.8%) for devices with PMA. Considering class I recalls, the probability of recall at 9 years is 0.9% (95% CI, 0.8%-1.0%) for devices with 510(k) clearance and 5.8% (95% CI, 2.8%-8.9%) for devices with PMA.

### Risk of Recall by Specialty

The specialty with the most devices was orthopedics (n = 5399), for which 24 devices were approved by PMA. Cardiovascular and radiology constituted the next largest specialty, with 3900 and 3577 devices, respectively. Cardiology was the specialty with the largest number of recalled devices with PMA (n = 115), with microbiology having the second largest number (n = 41).

**Figure 3.**
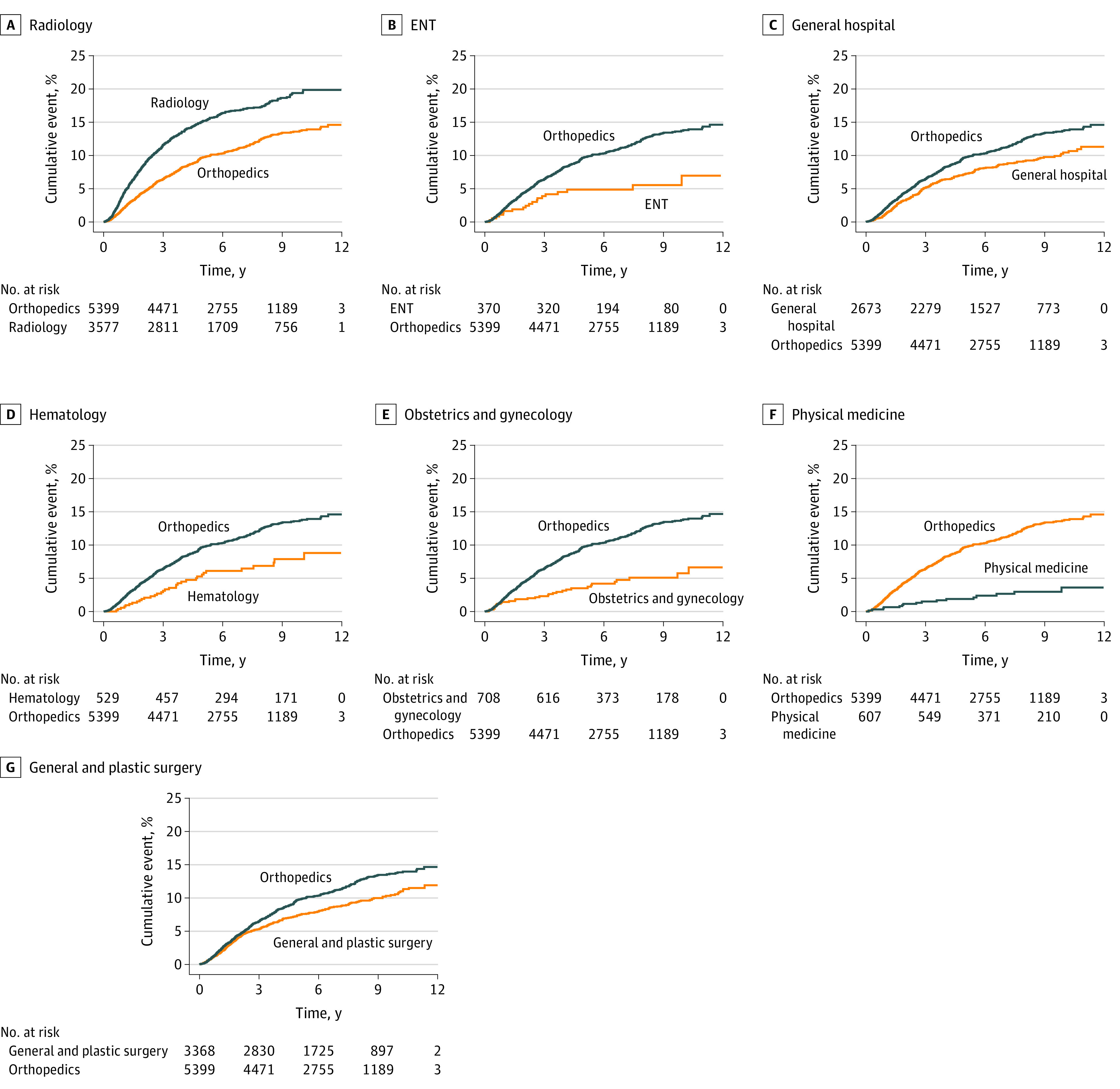
Time to First Recall by Medical Specialty Orthopedic surgery was chosen as the reference category. ENT indicates otolaryngology.

Only radiology (577 of 3577 devices [16.1%] were recalled) demonstrated a significantly increased risk of recall relative to the reference category (orthopedic surgery: 579 of 5399 devices [10.7%] were recalled), with a hazard ratio of 1.57 (95% CI, 1.32-1.87; *P* < .001). Six specialties had significantly lower recall than the reference category: general and plastic surgery (8.4% [284 of 3368]), otolaryngology (5.1% [19 of 370]), obstetrics and gynecology (4.4% [31 of 708]), physical medicine (2.6% [16 of 607]), hematology (6.2% [33 of 529]), and general hospital (8.4% [224 of 2673]) (Table 2 and Figure 3).

**Table 2.** All Recall and Class I Recall by Specialty and US Food and Drug Administration Pathway

Specialty^a^	Total No. (%) of devices for 510(k) and PMA	No. (%)^b^						HR of recall (95% CI)^d^	*P* value, Bonferroni adjusted
		510(k) Clearance			PMA				
		Total devices	Recalled devices	Devices with class I recalls^c^	Total devices	Recalled devices	Devices with class I recalls^c^		
Anesthesia	1201 (4.2)	1196 (99.6)	123 (10.2)	41 (3.4)	5 (0.4)	4 (0.3)	1 (0.1)	0.93 (0.7-1.25)	>.99
Cardiovascular	3900 (13.7)	3785 (97.1)	474 (12.2)	70 (1.8)	115 (3)	38 (1)	11 (0.3)	1.18 (0.98-1.42)	.11
Clinical chemistry	1456 (5.1)	1444 (99.2)	196 (13.5)	14 (1)	12 (0.8)	7 (0.5)	0	1.24 (0.97-1.58)	.14
ENT	370 (1.3)	367 (99.2)	18 (4.9)	2 (0.5)	3 (0.8)	1 (0.3)	0	0.47 (0.23-0.94)	.02
General and plastic surgery	3368 (11.8)	3350 (99.5)	282 (8.4)	13 (0.4)	18 (0.5)	2 (0.1)	1 (<0.1)	0.78 (0.62-0.96)	.008
General hospital	2673 (9.4)	2670 (99.9)	223 (8.3)	33 (1.2)	3 (0.1)	1 (<0.1)	0	0.75 (0.6-0.95)	.005
Gastrointestinal and urology	1485 (5.2)	1475 (99.3)	168 (11.3)	2 (0.1)	10 (0.7)	2 (0.1)	0	1.06 (0.82-1.38)	>.99
Hematology	529 (1.9)	529 (100)	33 (6.2)	5 (0.9)	0	0	0	0.56 (0.33-0.96)	.02
Immunology	338 (1.2)	335 (99.1)	28 (8.3)	0	3 (0.9)	0	0	0.7 (0.39-1.24)	>.99
Microbiology	759 (2.7)	718 (94.6)	81 (10.7)	6 (0.8)	41 (5.4)	8 (1.1)	0	1.02 (0.72-1.43)	>.99
Molecular genetics	4 (<0.1)	3 (75)	0	0	1	0	0	NA	NA
Neurology	1167 (4.1)	1156 (99.1)	90 (7.7)	13 (1.1)	11 (0.9)	3 (0.3)	1 (0.1)	0.75 (0.54-1.04)	.16
Obstetrics and gynecology	708 (2.5)	704 (99.4)	31 (4.4)	0	4 (0.6)	0	0	0.39 (0.23-0.68)	<.001
Ophthalmology	672 (2.4)	656 (97.6)	67 (10)	2 (0.3)	16 (2.4)	7 (1)	2 (0.7)	1.01 (0.7-1.46)	>.99
Orthopedics	5399 (18.9)	5375 (99.6)	578 (10.7)	6 (0.1)	24 (0.4)	1 (<0.1)	0	NA	NA
Pathology	112 (0.4)	80 (71.4)	14 (12.5)	2 (1.8)	32 (28.6)	8 (7.1)	0	1.36 (0.7-2.66)	>.99
Physical medicine	607 (2.1)	607 (100)	16 (2.6)	0	0	0	0	0.23 (0.11-0.48)	<.001
Radiology	3577 (12.5)	3565 (99.7)	575 (16.1)	6 (0.2)	12 (0.3)	2 (0.1)	0	1.57 (1.32-1.87)	<.001
Toxicology	231 (0.8)	231 (100)	15 (6.5)	1 (0.4)	0	0	0	0.58 (0.26-1.26)	.59
Total	28 556	28 246 (98.9)	3012 (10.5)	216 (0.8)	310 (1.1)	84 (0.3)	16 (0.1)	NA	NA

Abbreviations: ENT, otolaryngology; HR, hazard ratio; NA, not applicable; PMA, premarket approval.

^a^ Molecular genetics was excluded from analysis because it had only 4 devices.

^b^ All percentages are given with respect to the total number of devices within the specialty except the second column, which is with respect to the total number of devices included in the study (28 556).

^c^ Data on class I recalls for each specialty are included for reference but were not separately analyzed because of the relatively low numbers.

^d^ The HR describes the risk of any recall within the specialty compared with the reference category (orthopedic surgery).

## Discussion

Ensuring the safety of medical devices is integral to public health. Multiple authors have attempted to assess medical device safety by investigating recalls.^6,7,8,9,10,11,12^ To our knowledge, this is the largest study to date to analyze both all recalls and high-risk recalls, with 28 556 devices analyzed over 10 years. It is also the first study, to our knowledge, to account for the duration a device is on the market using a time-to-event analysis. These factors likely contributed to rejecting the hypothesis that devices with 510(k) clearance pose a greater risk of recall than those with PMA. Instead, we found that devices with PMA have 2.7 times the hazard of any recall and 7.3 times the hazard of class I recall when compared with devices with 510(k) clearance. At 9 years after device approval, the risk of any recall for devices with PMA and 510(k) clearance is 32% vs 13% and decreases to about 6% vs 1%, respectively, when considering only class I recalls. However, devices with 510(k) clearance comprise 99% of the devices to reach the market and comprised approximately 97% of both the 3096 devices recalled and the 5362 total number of recalls identified in the study period, making them a significant source of safety concern despite the devices demonstrating a lower risk of recall.

In hindsight, this finding may appear intuitive, considering that devices with PMA are high-risk devices by definition and are more commonly subject to postapproval studies and surveillance. Overall, however, the existing literature supports the opposite conclusion, with estimates ranging from twice to nearly 12 times the risk of recall for devices with 510(k) clearance compared with those with PMA.^6,11^ Similar findings are reported in the otolaryngology, ophthalmology, obstetrics and gynecology, and radiology literature.^8,9,10,12^

Most authors attribute their results to the clinical data required by the PMA; however, this runs counter to several studies showing significant shortcomings in the methods used for PMA pivotal trials.^6,7,8,9,10,11,12,19,20^ Rather, the discrepancy between the present study and other studies may be attributable to 2 major differences in methods. First, most previous literature analyzed the total number of recalls in a given period, not accounting for vastly larger numbers of devices with 510(k) clearance, time on the market, or devices having multiple recalls. Second, several authors likely included PMA supplements in their analysis, although they did not report it in their methods.^6,11^ Supplements have been shown to often rely on no, or sometimes weak, clinical data for approval, and their accumulation over time, reported to range from a median of 6.5 to 50 supplements per device, may lead to essentially unique devices from the original.^27,28,29^ The FDA, however, considers supplements only as modifications, and including them in a recall analysis dilutes the risk of recall by overestimating the actual number of devices with PMA.^24,25,26,27,29^ This finding may explain why Somberg et al^11^ reported nearly 6000 devices with PMA in 9 years with a 0.45% probability of class I recall compared with the present study, which found 310 devices in 10 years with a 6% risk of class I recall. Supplements may increase the risk of device recall; however, that research is beyond the scope of this investigation.

With regard to secondary end points, we found significant variation in the risk of recall between specialties. Although only radiology demonstrated a significantly increased risk of recall, 6 specialties had a significantly lower risk of recall than the reference category (orthopedic surgery). It is possible that the different proportions of high-risk devices within each specialty may have contributed to these findings. For instance, both physical medicine and hematology had no devices with PMA and had a significantly lower risk of recall. However, both pathology (28%) and cardiovascular (3%) had a higher percentage of devices with PMA, without increased risk of recall. Ghobadi et al^32^ investigated recall between specialties over a 14-year period and found similar variations, albeit mainly between different fields. They also identified physical medicine and obstetrics and gynecology devices as having very low risk of recall, although their analysis included only high-risk recalls. Other investigators have attempted to compare recall between fields as well but with varied results.^6,7,11^ The disparate findings between studies are likely attributable to the methodological differences noted previously.

Last, this study found 5362 recalls in the 12-year analysis period, with 97% for devices with 510(k) clearance and 3% for those with PMA. Zuckerman et al^7^ reported that 71% of high-risk recalls were for devices with 510(k) clearance, a number slightly lower than seen in this study, but they only examined recalled devices and did not count multiple recalls for the same device. To our knowledge, there is little existing literature analyzing devices with multiple recalls; however, the present study found that nearly one-third of recalled devices for both PMA and 510(k) clearance were recalled multiple times, with several devices in each group noted to have multiple class I recalls. This finding suggests that certain devices continue to raise safety concerns even after an initial recall, especially in those with multiple class I recalls. Caution, however, is urged in overinterpreting these results because the reasons for the subsequent recalls is unknown and beyond the scope of this investigation.

### Limitations

There are several important limitations to the study. First, several FDA policy changes were passed during the study period, such as the Medical Device User Fee Amendments, which increased funds that often support factory inspections and other surveillance activities, possibly leading to the discovery of more recalls. Ghobadi et al,^32^ however, found no significant increase in recalls associated with the passage of these amendments from 2007 to 2016. Next, recalls are highly dependent on adverse event reporting, particularly through the Manufacturer and User Facility Device Experience (MAUDE) database, which the FDA reports potentially includes inaccurate and incomplete data.^33^ Adverse events are also more likely to be identified for devices that are used more frequently, a confounder not readily adjusted for and not done in previous studies. Furthermore, a device can raise major safety concerns without being recalled. Two particularly publicized examples are the Bayer Essure device for permanent sterilization^34^ and laparoscopic morcellators,^35^ which have been linked to iatrogenic dissemination of potentially cancerous cells within the peritoneum. In neither case were recalls formally issued, but the FDA did publish multiple public updates and guidance documents. The Essure device was voluntarily withdrawn from the market by the manufacturer without a formal recall. If other devices were voluntarily withdrawn, they were not accounted for in this study or in most previous studies. This outcome is owing to inconsistent reporting in the FDA database and the fact that the FDA considers withdrawals to “involve a minor violation that would not be subject to legal action by the FDA.”^31^ Finally, recall is a surrogate end point, not necessarily capturing the patients’ level of pain or the extent of damage to public health. As an example, the Depuy ASR (Articular Surface Replacement) metal-on-metal hip implant recall, despite being a class II recall, has left a significantly larger impact on public health than the class I recall of the Echelon Flex Endopath Stapler (510(k) number: K081146), which cleared the 510(k) pathway the same year.^21,36^ These issues create an inability to capture all device-related safety concerns and are associated with a probable underestimation of the real problem, although we believe these results reflect the most accurate to date.

## Conclusions

In this cohort study, despite requiring clinical trials, the high-risk devices approved through PMA were associated with a substantially greater risk of recall than previously reported. Second, devices with 510(k) clearance accounted for most recalls, and the 13% risk of recall at 9 years likely underestimates safety problems, as previously noted. Although not specifically addressed in this study, we recognize the importance of balancing the need to ensure safety and effectiveness with bringing innovative treatments to patients as quickly as possible, and we join with multiple other authors in calling for increased postmarketing surveillance strategies to supplement the MAUDE database and Medwatch program. Health professionals can, and should, register device safety concerns with the FDA through Medwatch, although participation is voluntary.^11,29,37,38^ Recently implemented by the FDA is the Global Unique Device Identification Database, in which the FDA tracks devices with a unique device identifier.^39^ This identifier will eventually be linked to electronic health records and possibly with certain high-quality registries. We also agree with calls to increase the quality of evidence used in the pivotal trials performed for devices with PMA.^19,20,21,27,28,29^ Third, many unanswered questions remain regarding device safety, including the variability in recalls between specialties and individual devices, and further research is indicated to help safeguard the public health.
